# A Flow Cytometry-Based Approach for the Isolation and Characterization of Neural Stem Cell Primary Cilia

**DOI:** 10.3389/fncel.2018.00519

**Published:** 2019-01-14

**Authors:** Sara Monaco, Katja Baur, Andrea Hellwig, Gabriele Hölzl-Wenig, Claudia Mandl, Francesca Ciccolini

**Affiliations:** Interdisciplinary Center for Neurosciences (IZN), Department of Neurobiology, University of Heidelberg, Heidelberg, Germany

**Keywords:** primary cilium, ependymal cilium, subependymal zone, Sonic hedgehog, platelet-derived growth factor

## Abstract

In the adult mammalian brain, the apical surface of the subependymal zone (SEZ) is covered by many motile ependymal cilia and a few primary cilia originating from rare intermingled neural stem cells (NSCs). In NSCs the primary cilia are key for the transduction of essential extracellular signals such as Sonic hedgehog (SHH) and platelet-derived growth factor (PDGF). Despite their importance, the analysis of NSC primary cilia is greatly hampered by the fact that they are overwhelmingly outnumbered by the motile cilia. We here take advantage of flow cytometry to purify the two cilia types and allow their molecular characterization. Primary cilia were identified based on immunoreactivity to the marker adenylate cyclase type III (AC3) and differential levels of prominin-1 whereas motile cilia displayed immunoreactivity only to the latter. Consistent with the morphological differences between the two classes of cilia, enrichment of motile cilia positively correlated with size. Moreover, we observed age-dependent variations in the abundance of the two groups of ciliary organelles reflecting the changes associated with their development. The two cilia groups also differed with respect to the expression of signaling molecules, since PDGF receptor (PDGFR)α, smoothened (Smo) and CXC chemokine receptor (CXCR)4 were only detected in isolated primary but not motile cilia. Thus, our novel method of cilia isolation and characterization by flow cytometry has the potential to be extended to the study of cilia from different tissues and organs, providing a powerful tool for the investigation of primary cilia in physiological and pathological conditions.

## Introduction

Primary cilia have an emerging function in the transduction of developmental and homeostatic pathways and their dysfunction is associated with a number of human diseases, collectively referred to as ciliopathies (Berbari et al., 2009; Tobin and Beales, 2009). Primary cilia are present throughout the brain and they are involved in several functions including neurogenesis, migration, autophagy and development (Guemez-Gamboa et al., 2014). The adult subependymal zone (SEZ) is the largest germinal niche in the adult brain, where neural stem cells (NSCs) mainly generate new interneurons for the olfactory bulb throughout adulthood. The apical side of the SEZ is lined with a myriad of motile cilia stemming from ependymal cells and a few primary cilia protruding from NSCs. The two cell types form a characteristic pinwheel structure at the apical SEZ surface in which ependymal cells encircle a NSC (Mirzadeh et al., 2008). Like adult NSCs, ependymal cells are generated from radial glia precursors perinatally and continue to develop during the first weeks after birth (Merkle et al., 2004; Spassky et al., 2005). As a consequence, primary cilia extending from radial glia represent the prevailing cilia type before birth. Both ependymal cells and NSCs undergo changes during postnatal aging. In particular, pinwheel structures become rarer in aged mice (Shook et al., 2012) likely due to NSCs losing the apical attachment (Obernier et al., 2018). Ageing also affects the ependymal layer with loss of ependymal cells into the ventricular space (Del Bigio, 2010). Primary and motile cilia are defined by striking morphological differences. The primary cilia axoneme, which is 1–9 μm long (Dummer et al., 2016) and 0.2–0.3 μm wide, is constituted at its core by a ring of nine microtubule pairs (9 × 2 + 0). In contrast, the generally longer motile cilia, whose length is extremely variable in different tissues (Lee, 2013), have a 9 × 2 + 2 core structure with the ring of external microtubule doublets connected by inner and outer dynein arms and an additional central pair of microtubule singlets. Despite being structurally and functionally different both primary and motile cilia express prominin-1, a glycoprotein commonly used to isolate stem and progenitor cells from the developing and adult nervous system, which is selectively localized in membrane protrusions (Weigmann et al., 1997), including cilia (Pfenninger et al., 2007; Coskun et al., 2008). However, whereas prominin-1 expression is a constant feature of ependymal motile cilia, a subset of primary cilia in the SEZ lacks the expression of the glycoprotein at the cell membrane (Codega et al., 2014; Khatri et al., 2014). Modification of the tubulin residues such as acetylation and glycosylation, which increase the stability of the microtubule and the length of the axoneme, are found in both types of cilia. However, the type 3 adenylate cyclase (AC3) localizes to primary cilia only and it is considered to be a marker of primary cilia in all regions of the mouse brain (Bishop et al., 2007).

The beating of ependymal motile cilia contributes to the movement of the cerebrospinal fluid (CSF) in the ventricular system of the brain representing an essential component of a protecting barrier whose integrity is important to maintain the size of the ventricles (Shook et al., 2014) as well as to create concentration gradients for the guidance of migrating neurons (Sawamoto et al., 2006). Underscoring the importance of the ependymal ciliary function, several neurological conditions (Ikeda et al., 2005; King, 2006; Suzuki et al., 2009) such as hydrocephalus (Lee, 2013; Jiménez et al., 2014) and schizophrenia (Palha et al., 2012) have been associated to impaired circulation of the CSF. The flow of the CSF is also disrupted in Huntington’s disease, which leads to an increase in the length of ependymal cilia (Keryer et al., 2011). In contrast, primary cilia in radial glia have been associated to the regulation of cell cycle progression (Tong et al., 2014; Izawa et al., 2015). This function reflects the fact that the mother centriole in the basal body of the primary cilia is needed for the mitotic spindle formation. Furthermore, the organelle is essential to transduce signals involved in the regulation of progenitor proliferation such as Sonic hedgehog (SHH) and platelet-derived growth factor (PDGF) signaling (Youn and Han, 2018). Although genes coding for ciliary function are specifically enriched in NSCs and cilia depletion affects NSC quiescence in the SEZ (Beckervordersandforth et al., 2010), not all quiescent NSCs in this region display a primary cilium (Khatri et al., 2014). Moreover ablation of primary cilia affects proliferation only in NSCs of the ventral SEZ (Khatri et al., 2014; Tong et al., 2014). Thus, the function of primary cilia in NSCs is still unclear.

The direct analysis of the expression of signaling molecules in primary cilia would significantly contribute to elucidate their function. Here we describe an innovative flow cytometry-based method to isolate motile and primary cilia from the SEZ and provide evidence for its suitability to analyze the molecular composition of cilia both at the population as well as at the single cilia level.

## Materials and Methods

### Analysis and Purification of Cilia From the SEZ by Flow Cytometry

#### Deciliation and Immunostaining

All animal experiments were approved by the Regierungspräsidium Karlsruhe and the local authorities of Heidelberg University. Adult mice were killed by CO_2_ inhalation followed by cervical dislocation whereas E18 embryos were sacrificed by decapitation. The brain was removed from the skull and the SEZ was dissected in ice-cold dissection medium (150 μM sucrose, 125 μM NaCl, 3.5 mM KCl, 1.2 mM NaH_2_PO4, 2.4 mM CaCl_2_, 1.3 mM MgCl_2_, 2 mM HEPES, 6.65 mM D-(+) glucose; Khatri et al., 2014). The dissected tissue was put in sort medium (NS-A basal medium and L15 medium (1:1), 2% B27 supplement, 1% fetal calf serum (FCS), 0.6% D-(+) glucose, 10 ng/ml huFGF-2, 0.001% DNase) containing APC-conjugated anti-prominin-1 antibody (Becton Dickinson, BD) for 30 min at 4°C and then washed at room temperature with sort medium to eliminate the excess of antibody. Deciliation was performed by combining the two most common methods for cilia detachment: mechanical shear and calcium shock (Mitchell et al., 2009). Briefly, the tissue was incubated in sort medium containing 10 mM CaCl_2_ and subjected to mechanical agitation on a rotatory shaker (200 rpm) at 4°C. After 20 min, the medium, containing cilia, was centrifuged for 1 min at 2,000 rpm at 4°C to remove cellular debris. The supernatant was collected and immunostained with anti-AC3 antibodies (Santa Cruz) and Alexa Fluor 488-conjugated secondary antibodies for 30 min at 4°C.

#### Fluorescence Activated Cell Sorting

Cilia preparations were sorted on a FACSAria II cytometer (BD) at single event precision. Sorting gates were set based on florescence levels of samples stained with secondary antibodies only or samples which were not incubated with any antibodies (autofluorescence).

To standardize Forward (FSC) and Side (SSC) scatter values we used as reference beads of known size, i.e., 3 μm (Rainbow Fluorescent Particles, BD) and 6 μm (Accudrop beads, BD). Sorted ciliary particles that based on the reference beads had a size smaller than 3 μm, between 3 μm and 6 μm or greater than 6 μm were separately collected and analyzed. Hereafter they will be referred to as 2 μm (<3 μm), 5 μm (3–5 μm) and 8 μm (≥6 μm) particles.

#### Characterization of the Cilia Types (Primary vs. Motile)

For each size group, double staining with anti-AC3-Alexa 488 and prominin-APC antibodies was analyzed and four gates were set: AC3^+^/Prom^−^ (AC3^+^), AC3^+^/Prom^+^ (double positive, DP), AC3^−^/Prom^+^ (Prom^+^), AC3^−^/Prom^−^ (double negative, DN).

#### Functional Characterization

For each size group immunostaining was used for detecting one of the following antigens: Smoothened, PDGFRα or CXC chemokine receptor (CXCR)4. For PDGFRα and CXCR4 staining, the dissected tissue was incubated in sort medium containing anti-prominin-APC (Miltenyi Biotec) and anti-PDGFRα or CXCR4 antibodies conjugated with PE (Invitrogen) for 30 min and washed with sort medium before proceeding to deciliation and staining with anti-AC3 antibody (Invitrogen) manually conjugated with Dylight 488 (Abcam, cat# ab201799). For Smoothened analysis, the tissue fragments were incubated with or without SAG (Cayman Chemical, cat# 11914, 200 nM) in NS-A medium (Euroclone/Biozol) containing 2 mM L-glutamine (Gibco), 100 U/ml penicillin/streptomycin (Gibco), 2% B27 supplement (Invitrogen), 10 ng/ml huFGF-2 (Peprotech) overnight at 37°C. The samples were labeled with anti-Smoothened antibodies (Novus Biologicals) and anti-rabbit-APC secondary antibodies for 30 min. The samples were then washed, deciliated and the supernatant was subjected to AC3 staining as described above.

### Western Blot

For western blot analysis, a specific number of particles was sorted into PBS containing proteinase inhibitor and after ultracentrifugation at 4°C at 27,000× *g* for 40 min resuspended in RIPA buffer. The samples were subjected to standard immunoblot analysis with mouse anti-acetylated tubulin antibody (Sigma-Aldrich). Immunoreactivity was quantified using ImageJ and the results were normalized by the number of particles collected in each sample.

### Whole Mount Immunostaining

Whole mount dissection was performed as previously described (Mirzadeh et al., 2008). The dissected tissue was fixed in 3% formaldehyde/4% sucrose (dissolved in PBS) for 2 h, permeabilized with 0.5% NP-40 for 10 min, incubated in 100 mM glycine to inactivate residual aldehyde groups, and blocked in 5% FCS for 1.5 h. The samples were then incubated with primary antibodies against acetylated tubulin (Sigma Aldrich) and either β-catenin (Santa Cruz), AC3 (Invitrogen) or prominin-1 (kind gift from Denis Corbeil, Technical University of Dresden) in 0.1% NP-40 over night at 4°C. After washing, the samples were incubated with secondary antibodies and DAPI for nuclear counterstain for 2 h and analyzed using a C2 Plus confocal microscope with NIS software (Nikon) or a TCS SP8 confocal microscope with LAS X software (Leica).

### Antibodies

A list of all primary antibodies used, source, catalog/lot number and concentration is provided in Supplementary Table S1.

### Electron Microscopy

Scanning electro microscopy (SEM) was performed as previously described (Khatri et al., 2014). Briefly, the samples were fixed with 2% glutaraldehyde in 0.1 M sodium phosphate buffer. After washing and postfixation with 2% osmium tetroxide/1.5% potassium ferrocyanide for 1 h, they were washed and dehydrated with an ascending series of ethanol and pure acetone before critical point drying. The samples were then sputter-coated with an 80% gold, 20% palladium alloy and examined with a ULTRA 55 field-emission scanning electron microscope (ZEISS).

### Statistical Analysis

Statistical significance tests (ANOVA with Tukey’s *post hoc* test or Student’s *t*-test) of at least three independent experiments were calculated using GraphPad Prism and OriginPro 2016. Data represent means ± SEM. *P*-values are indicated in the figures as follows: *p < 0.05, **p < 0.01, ***p < 0.001, ****p < 0.0001.

## Results

### A Flow Cytometry-Based Method for Isolation of Primary Cilia From the SEZ

**Figure 1:**
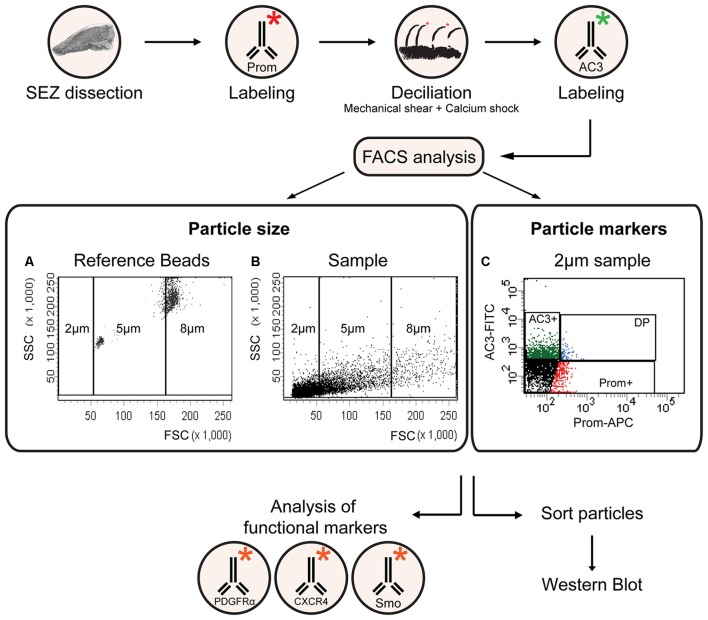
A flow cytometric approach for the isolation and characterization of primary and motile cilia. Flowchart of the experimental procedure for cilia identification and characterization according to size and marker expression. The * symbol represents a conjugated fluorophore. **(A,B)** FACS plots showing the distribution of reference beads and a sample of cilia according to forward (FSC) and side (SSC) scatter. **(C)** Representative FACS plot illustrating the distribution of 2 μm particles upon immunostaining with antibodies directed against adenylate cyclase type iii (AC3; AC3-FITC) and prominin-1 (Prom-APC). The gates indicate AC3 positive (AC3+), Prominin positive (Prom+) and double positive (DP) particles.

**Figure 2:**
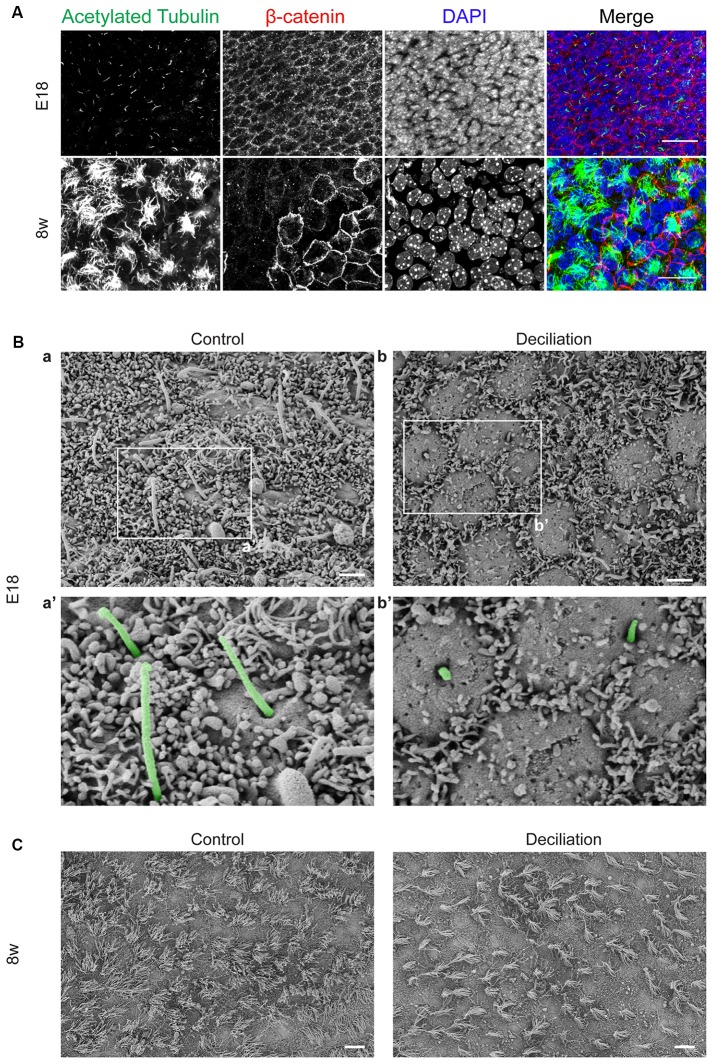
Primary and motile cilia on the apical side of the subependymal zone (SEZ). **(A)** Confocal images showing a representative example of the apical side of the SEZ in whole mount preparations immunostained for acetylated tubulin (green) and β-catenin (red). Nuclei were visualized by DAPI staining (blue). Scale bars: 20 μm. **(B)** Scanning electron microscope (SEM) images of the apical surface of the lateral ventricular wall in whole mount preparations from E18 mice before (Control) and after deciliation. Higher magnification views of panels **(a,b)** are shown in **(a’,b’)**, respectively. Primary cilia are highlighted in green. After the treatment many of the primary cilia are detached and only short stumps are left on the cell surface **(b’)**. Scale bars: 2 μm. **(C)** SEM images of the apical surface of the SEZ in whole mount preparations of 8 week-old mice (8w). Scale bars: 10 μm.

**Figure 3:**
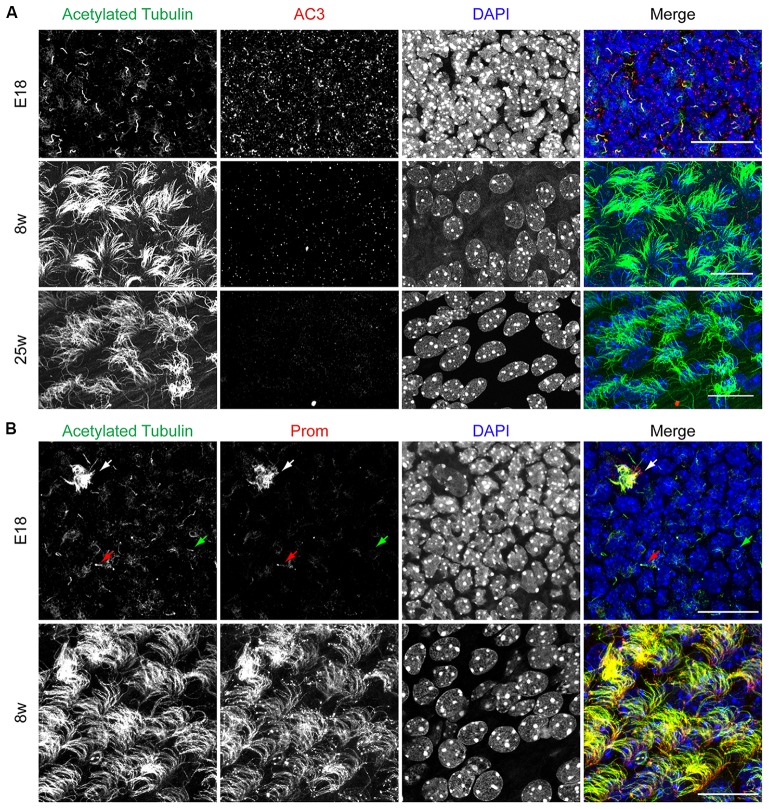
AC3 and prominin-1 expression on the apical side of the SEZ. **(A)** Representative confocal images illustrating the apical side of the SEZ upon immunostaining with acetylated tubulin (green) and AC3 (red). Nuclei were visualized by DAPI. Scale bar: 20 μm. **(B)** Confocal images of whole mount preparations immunostained for acetylated tubulin (green) and prominin-1 (Prom, red). Nuclei were visualized by DAPI. White arrow indicates motile cilia, red arrow indicates Prom^+^ primary cilia and green arrow indicates Prom^−^ primary cilia. Scale bars: 20 μm.

**Figure 4:**
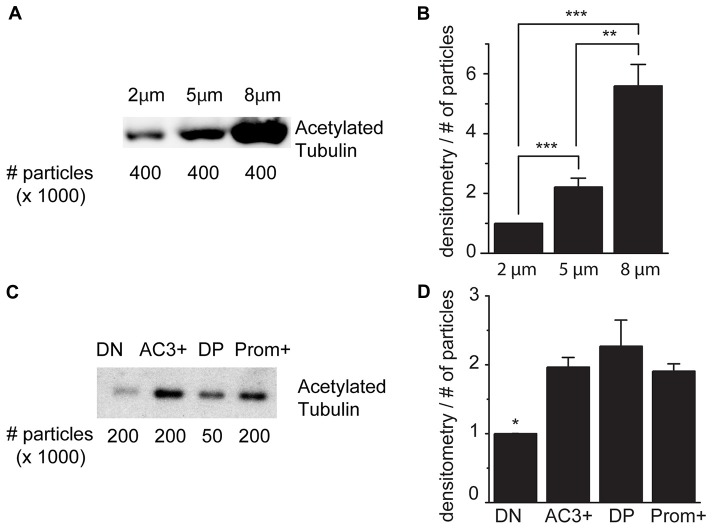
Acetylated tubulin expression in sorted cilia. **(A)** Representative immunoblot analysis of acetylated tubulin in samples of sorted particles of different sizes from adult mice. **(B)** Densitometric analysis of four independent experiments. **(C)** Immunoblot analysis of acetylated tubulin in samples sorted according to the expression of AC3 and prominin-1: double negative (DN), AC3^+^ only (AC3^+^), double positive (DP), prominin-1 only (Prom^+^). **(D)** Densitometric analysis of three independent experiments normalized for the number of particles. Values represent the fold increase relative to the fist sample. Error bars represent SEM. Statistically significant differences are indicated with asterisks (*p < 0.05, **p < 0.01, ***p < 0.001).

As schematically illustrated in Figure 1, we took advantage of flow cytometry to set up a novel approach to identify and isolate primary cilia from the murine SEZ. We performed this method with samples obtained from the SEZ of adult mice, in which motile ependymal cilia are much more abundant than primary cilia, and the corresponding germinal area dissected from embryos at embryonic day (E) 18, when primary cilia represent the vast majority of the cilia. Such age-dependent differences in cilia type proportion are readily visible upon immunostaining of the apical side of the germinal area at the two ages with antibodies directed against acetylated tubulin, which is expressed in both types of cilia (Supplementary Table S2; Figure 2A). Critical steps of the procedures are the deciliation, i.e., the detachment of cilia from the cell body, the identification and the separate collection of the two cilia types. To promote deciliation we used the traditional method of mechanical shearing in the presence of a high calcium ion concentration (Hastie et al., 1986; Mitchell et al., 2009; Ishikawa and Marshall, 2013). Comparing SEM images of the apical surface of the lateral ventricle wall of E18 (Figure 2B) and adult mice (Figure 2C), after deciliation or no treatment (Control), showed that the treatment was effective in removing primary but not motile cilia. In untreated embryonic tissue, primary cilia were readily visible (Figures 2Ba,a′, highlighted in green) whereas, after deciliation, they were largely absent (quantification in Supplementary Figure S1A). Sometimes a remaining stump could be detected (Figures 2Bb,b′, highlighted in green). This suggests that deciliation also gives rise to fragments of different sizes, as previously reported (Mitchell et al., 2009). In contrast, many tufts of ependymal cilia were still present after deciliation, (Figure 2C) although their number was significantly reduced (quantification in Supplementary Figure S1B). In order to distinguish motile from primary cilia we have taken advantage of differential expression of prominin-1 (Prom) and AC3 in the two cilia types. As summarized in Supplementary Table S2, whereas prominin-1 in the adult tissue is expressed more strongly in ependymal cells, AC3 has only been detected in primary cilia (Bishop et al., 2007). Consistent with this, immunostaining of AC3 in whole mount preparations of the SEZ of mice of different ages revealed AC3 expression in primary but not motile cilia and that the immunostaining decreased with age (Figure 3A). On the contrary, prominin-1 expression was detected in both cilia groups (Figure 3B). However, whereas in motile cilia prominin-1 was expressed at consistently high levels (Figure 3B, white arrow), primary cilia displayed high variability in expression levels, ranging from very high (Figure 3B, red arrow) to undetectable (Figure 3B, green arrow). To address variability in cilia length, ranging between 1 μm and 9 μm (Dummer et al., 2016), we have used beads of known size to set the forward (FSC) and side light scatter (SSC) parameters (Figures 1A,B) and monitor changes in particle size during sorting. Although for bigger particles, like for example cells, the first parameter reflects cell size whereas the second indicates complexity (granularity), for smaller particles the measurement of both parameters provides a better detection of changes in size. Independent of size, we detected four types of particles: primary cilia particles immunopositive for either AC3 only (AC3^+^) or double immunopositive (DP) for both AC3 and Prom, motile cilia particles immunopositive for Prom only (Prom^+^) and double immunonegative fragments (DN; Figure 1C). To further investigate their nature, the four populations of particles were separately collected by FACS, concentrated by ultracentrifugation and then analyzed by western blot to investigate the expression of acetylated tubulin (Figure 4). Particles were first sorted based on differences in size only (Figure 4A). After normalization according to the number of sorted particles, quantitative analysis of the western blots showed that although acetylated tubulin was present in all sorted populations, its amount increased with particle size (Figure 4B). This corroborates that the sorted material indeed contains cilia. We next sorted the particles based on differential staining. We collected the same number of particles (200,000) for DN, AC3^+^ and Prom^+^ particles but only 50,000 of the less abundant DP particles (Figure 4C). Densitometric analysis after normalization for the number of particles revealed that AC3^+^, DP and Prom^+^ populations similarly contain at least double the amount of acetylated tubulin than DN particles (Figure 4D). This indicates that the three groups of immunopositive particles indeed contain cilia material whereas the DN particles include contaminating material and a few ciliary fragments.

### Flow Cytometric Analysis of Cilia Markers AC3 and Prominin-1 Allows Identification of Primary and Motile Cilia

**Figure 5:**
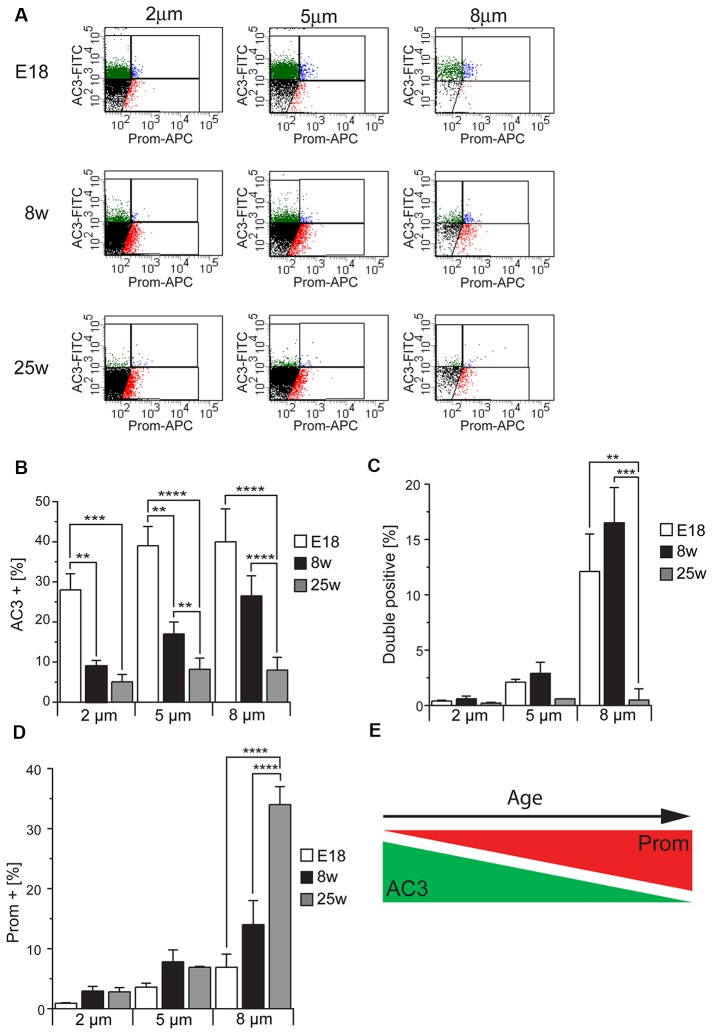
Cilia characterization according to the expression of AC3 and prominin-1 in different mouse ages. **(A)** Representative FACS plots illustrating the distribution of particles of different sizes according to AC3 and prominin-1 (Prom) staining in E18 embryos, 8 week-old (8w) and 25 week-old (25w) mice. **(B–D)** Quantification of AC3^+^, DP and Prom^+^ particles in at least three independent experiments. Values represent the average percentage of particles in each population. Error bars represent SEM. Statistically significant differences are indicated with asterisks (**p < 0.01, ***p < 0.001, ****p < 0.0005). **(E)** Schematic representing the relative expression of AC3 and Prom across ages.

To further validate our method for the identification of primary and motile cilia by flow cytometry, we took advantage of the fact that before birth motile cilia are very rare whereas primary cilia in the SEZ decrease with aging (Figure 2A). Therefore, we next used flow cytometry to investigate the abundance of the three cilia-enriched fractions defined by the differential antigen expression and subdivided according to size in preparations obtained either from E18 embryos or 8 week or 25 week-old mice (Figure 5). As illustrated by representative FACS plots (Figure 5A), at each age and for each size we measured the same number of total particles. At all ages examined collected cilia consisted mostly of 2 μm particles followed in decreasing order of abundance by 5 μm and 8 μm particles (Supplementary Figure S2). This is probably due to the fragmentation of the cilia during the process of deciliation (illustrated in Figure 2Bb′). Quantitative analysis of the number of immunopositive particles in each size-defined population highlighted that the abundance of AC3^+^ (Figure 5B) and DP (Figure 5C) particles greatly declined with age. However, whereas the number of AC3^+^ particles progressively declined with age, the number of DP particles did not vary significantly between samples obtained from E18 embryo and 8-week-old mice, but abruptly decreased thereafter. Moreover, whereas at all ages AC3^+^ particles were similarly distributed across the various size groups (Figure 5B), DP cilia were mostly found in the 8 μm subpopulation. These observations suggest that the AC3^+^ and DP particles represent distinct types of primary cilia, which is consistent with the variability of prominin-1 expression observed in primary cilia by immunostaining (see Figure 3B). In contrast to primary cilia, the fraction of Prom^+^ particles increased with age, reaching its maximum level in preparations of 25-week-old mice (Figure 5D). Moreover, in samples obtained from adult but not E18 mice this group was particularly enriched in the 8 μm fraction, consistent with the fact that motile cilia reach full length after birth and are generally longer than primary cilia.

Taken together, these results show that the number of AC3^+^ particles is highest in the embryonic preparations and progressively decreases in samples obtained from older mice in contrast to Prom^+^ particles, which increase with age (Figure 5E). Since apical primary cilia in the SEZ are present on NSCs, the observed age-dependent decline in primary cilia particles is consistent with previous findings showing that the neurogenic capacity of the SEZ declines with age due to a progressive decrease in NSCs (Maslov et al., 2004; Luo et al., 2006; Bouab et al., 2011; Capilla-Gonzalez et al., 2014).

### Analysis of PDGFRα and CXCR4 Expression in Sorted Particles

**Figure 6:**
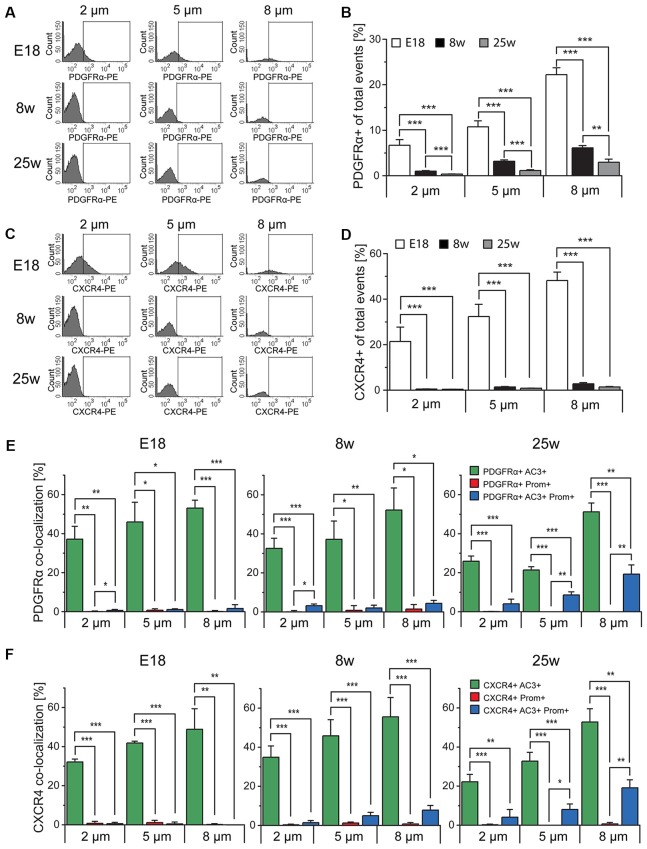
Platelet-derived growth factor receptor α (PDGFRα) and CXC chemokine receptor 4 (CXCR4) expression in primary cilia. **(A,C)** FACS histograms depicting PDGFRα and CXCR4 total expression in particles of different size in E18 embryos (E18), 8 week-old (8w)- and 25 week-old (25w) mice. The gates include PDGFRα-PE or CXCR4-PE positive particles. **(B,D)** Quantification of the average percentage of PDGFRα^+^ and CXCR4^+^ particles in at least three independent experiments. **(E,F)** Quantitative analysis of the co-localization of PDGFRα and CXCR4 with AC3 (green bars), prominin-1 (Prom; red bars) and both AC3 and Prom (blue bars), as percentage of PDGFRα^+^ or CXCR4^+^ total particles. Values represent the average percentage of particles in each population. Error bars represent SEM. Statistically significant differences are indicated with asterisk (*p < 0.05, **p < 0.01, ***p < 0.001).

To further confirm the identity of the sorted particles as primary or motile cilia, we next investigated the expression of signaling molecules which have been found in primary cilia, such as PDGFRα and CXCR4 (Supplementary Table S2; Schneider et al., 2005; Busillo and Benovic, 2007; Christensen et al., 2017; Schmid et al., 2018). To this end we obtained cilia preparations from the tissue of E18, 8 week and 25 week-old mice and analyzed particles of each given size either for PDGFRα (Figures 6A,B) or CXCR4 (Figures 6C,D) immunoreactivity. This analysis revealed that, independent of the size of the particles, the expression of both receptors drastically decreased after birth and continued to decrease with age in the case of PDGFRα. In contrast, CXCR4 was hardly detectable in cilia obtained from adult mice and no significant difference was observed between the two age groups (Figures 6A–D). However, independent of the age there was a positive correlation between the size and the relative expression of both antigens, which may indicate discontinuous expression of the two receptors along the cilia, leading to absent immunoreactivity in some of the fragments. We next investigated similar cilia preparations after triple immunostaining with antibodies specific for prominin-1 and AC3 and either PDGFRα or CXCR4 (Figures 6E,F). This analysis revealed that in every sub-population of size and age, almost all the particles displaying PDGFRα (Figure 6E) or CXCR4 (Figure 6F) immunoreactivity had also a marker profile of primary cilia, i.e., AC3^+^ and DP and not of Prom^+^ motile cilia. Indeed, depending on size and age, this fraction represented between 0% and 0.7% of the labeled particles. Moreover, although at every age analyzed the majority of the cilia immunoreactive for either receptor were AC3^+^, with increasing age the abundance of PDGFRα^+^ and CXCR4^+^ cilia displaying a DP profile significantly increased, suggesting that not only the number but also the type of primary cilia expressing either receptor changes with age.

### Analysis of Smoothened Expression in Sorted Particles

**Figure 7:**
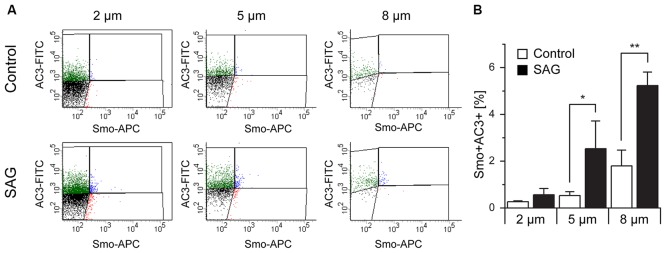
Co-localization of Smoothened and AC3 in primary cilia. **(A)** Representative dot plots of untreated (Control) and SAG-treated samples (SAG) from E18 embryos immunostained for AC3-FITC and Smoothened-APC (Smo-APC). **(B)** Quantification of the percentage of Smo^+^AC3^+^ particles. Bar graphs show mean ± SEM, *p*-values are calculated with Student’s *t*-test. Statistically significant differences are indicated with asterisks (*p < 0.05, **p < 0.01).

Another protein known to be associated with primary cilia is Smoothened (Smo; Supplementary Table S2) which, upon binding of SHH to Patched1, translocates to the primary cilium (Corbit et al., 2005; Eggenschwiler and Anderson, 2007; Rohatgi et al., 2007; Wilson et al., 2009). To activate SHH signaling, we incubated E18 tissue fragments in medium containing smoothened agonist SAG (Bijlsma et al., 2012; Fan et al., 2014; Lewis and Krieg, 2014; Milenkovic et al., 2015) and analyzed the effect of the treatment on Smo expression in primary cilia (Figure 7A). Quantitative analysis of AC3 and Smo co-expression showed that, independent of the particles size, exposure to SAG increased the expression of Smo within the population of AC3^+^ primary cilia, especially in the subpopulation of bigger (8 μm) particles (Figure 7B). This observation is consistent with the notion that activation of Smo leads to its translocation to the primary cilium and it further confirms the identity of the AC3^+^ particles as primary cilia.

## Discussion

Primary cilia have recently come to the forefront of the scientific interest after the discovery that a wide range of human diseases, collectively referred to as ciliopathies, are caused by defective functions of these organelles (Waters and Beales, 2011). For cilia purification and characterization, we took advantage of flow cytometry, which unlike the traditional approach of purification in sucrose density gradient (Raychowdhury et al., 2005; Mitchell et al., 2009) allows combining high throughput characteristics with highly sensitive analysis of multiple parameters in single particles of interest. Because of the analytical power of flow cytometry we were able to distinguish primary from motile cilia and investigate variability within these cilia types. For the differential identification of cilia we exploited the fact that in the brain AC3 is abundantly expressed in primary cilia, but not in motile cilia of the ependymal layer (Bishop et al., 2007). A possible exception may be represented by the cilia lining the 3rd ventricle, which express AC3 (Chen et al., 2016) however it was not investigated whether these organelles are motile cilia or primary cilia derived from tanycytes (Jarvis and Andrew, 1988; Mirzadeh et al., 2017). Consistent with the notion that it is present in primary but not motile cilia, we showed here that AC3 readily stains the primary cilia of embryonic radial glia but not the motile cilia in the adult SEZ. We here found that the majority of primary cilia do not express prominin-1, which is in keeping with previous observations in the adult SEZ (Codega et al., 2014; Khatri et al., 2014). Although prominin-1 was found widely expressed in embryonic radial glia, the expression was often associated with the apical membrane and not only with primary cilia (Dubreuil et al., 2007). Besides marker expression, several lines of evidence support our conclusion that the approach allows to discriminate primary vs. motile cilia. First, we found that the abundance of the two groups varies during aging reflecting the known changes in the presence of apical progenitors (Maslov et al., 2004) and ependymal cells (Capilla-Gonzalez et al., 2014). Second, the expression of the investigated signaling molecules was only observed in the group of primary cilia particles. In neural precursors several receptor-dependent pathways require a functioning primary cilium (Guemez-Gamboa et al., 2014). Despite the mechanism being still unclear, it is well established that SHH stimulation leads to increased presence of Smoothened in the primary cilium (Corbit et al., 2005; Rohatgi et al., 2007). Our data that PDGFRα localizes to primary cilia is also consistent with previous observations showing that neural precursors depend on primary cilia for transduction of PDGFRα signaling (Carter et al., 2012) and indicate that, like in fibroblasts (Schneider et al., 2005), also in neural precursors PDGFRα localizes to the primary cilium. Both CXCR4 and CXCR7 have been shown to localize to the cilia of developing interneurons (Wang et al., 2011). In the postnatal brain CXCR4 has been shown to be present also in neural progenitors of the SEZ (Tran et al., 2007). Moreover CXCR4-mediated signaling affects homing, (Kokovay et al., 2012), proliferation (Wu et al., 2009) and migration (Imitola et al., 2004; Carbajal et al., 2010) of neural precursors in culture and *in vivo*. Since the expression of CXCR4 has been shown throughout the ependymal layer of the SEZ (Stumm et al., 2002) our data suggest that the receptor does not localize to the motile cilia. We also found that the expression of all signaling molecules analyzed was particularly pronounced in AC3 single positive but not DP primary cilia. This may reflect the different nature of the two ciliary particles. Indeed, the nature of the population expressing PDGFRα in the adult SEZ is still a matter of debate (Jackson et al., 2006; Chojnacki et al., 2011) and it is known that NSCs more differentiated precursors, but not ependymal cells respond to SHH in the adult SEZ (Ahn and Joyner, 2005). The fact that the expression of both PDGFRα and CXCR4 declines with age especially in AC3 single positive particles further supports the hypothesis of two distinct groups of primary cilia. However, further analysis would be necessary to conclusively address this issue.

Further evidence that we could distinguish between motile and primary cilia is the fact that the deciliation method used here allowed us to enrich for the latter organelle type. An increase in the intracellular concentration of calcium was first used for the detachment of flagella from lower eukaryotes (Watson and Hopkins, 1962; Gibbons, 1965; Hansma and Kung, 1975; Adoutte et al., 1980). In these studies exposure to high calcium ion concentration, often in the presence of detergents, was used to elicit the lysis of the membranous fraction and the detachment of the flagella at the level of the transition zone. Intracellular calcium elevations were shown to trigger shedding of flagella in protists by a mechanism that involves microtubule severing activity and contraction of the fibers in the transition zone, which may also lead to membrane fission (Quarmby and Hartzell, 1994; Sanders and Salisbury, 1994; Lohret et al., 1998). Calcium influx leads to deciliation also of olfactory (Anholt et al., 1986) and primary cilia (Overgaard et al., 2009). The relative inefficacy of the approach on the detachment of the motile cilia likely reflects the different expression of molecules regulating calcium homeostasis and sensing in motile cilia compared to primary cilia as well as flagella (Satir and Christensen, 2007; Delling et al., 2013; Doerner et al., 2015; Inaba, 2015; Lishko and Kirichok, 2015). The method triggers membrane fusion after deciliation, thereby minimizing contamination from cellular material (Satir et al., 1976). Nevertheless, contaminating cellular material including mitochondria was often observed in classical preparations. In addition, osmotic lysis of the membrane leading to the separation of the skeletal axoneme from the ciliary membrane was often observed. In contrast, the particles we purify in the present study likely contain both cilium components as for their purification we used antibodies binding to integral or peripheral proteins of the ciliary membrane and by western blot analysis we observed an enrichment in acetylated tubulin, which is found in the axoneme. However, the fact that we observed acetylated tubulin not associated with ciliary membrane proteins, i.e., in the DN particles, may indicate the presence of ciliary axoneme structures dissociated from the membrane.

Thus, our novel flow cytometry-based approach for the isolation of primary cilia is a new tool for the investigation of these organelles whose function is still poorly understood.

## Author Contributions

SM and KB: data collection and analysis, manuscript writing. AH: data collection, manuscript editing. GH-W and CM: data collection. FC: conception and design and overall data interpretation, manuscript writing.

## Conflict of Interest Statement

The authors declare that the research was conducted in the absence of any commercial or financial relationships that could be construed as a potential conflict of interest.

## Supplementary Material

FIGURE S1 Quantification of primary and motile cilia on SEM images. **(A)** Quantification of the number of primary cilia in four SEM fields of SEZ whole mounts of E18 mice before (control) and after deciliation. **(B)** Quantification of the number of tufts of motile cilia in three SEM fields of SEZ whole mounts of 8 week-old mice (8w) before and after deciliation. Bar graphs show mean ± SEM, *p*-values are calculated with Student’s *t*-test. (**p* < 0.05, ****p* < 0.001).

FIGURE S2 Sorting of particles according to size. Quantification of the relative abundance of sorted particles of different sizes in embryo (E18) 8 (8w) and 25 (25w) week-old mice from three independent experiments. Values represent the average percentage of particles in each population.

TABLE S1 List of antibodies used in this study.

TABLE S2 List of markers used in this study to identify and characterize primary and motile cilia.
