# Supplementary material for: A Flow Cytometry-Based Approach for the Isolation and Characterization of Neural Stem Cell Primary Cilia
Source: Front Cell Neurosci. 2019 Jan 14;12:519. doi: 10.3389/fncel.2018.00519 (PMC6339872; doi:10.3389/fncel.2018.00519)
Supplement: FIGURE S1 — Quantification of primary and motile cilia on SEM images. (A) Quantification of the number of primary cilia in four SEM fields of SEZ whole mounts of E18 mice before (control) and after deciliation. (B) Quantification of the number of tufts of motile cilia in three SEM fields of SEZ whole mounts of 8 week-old mice (8w) before and after deciliation. Bar graphs show mean ± SEM, p-values are calculated with Student’s t-test. (*p < 0.05, ***p < 0.001). [file Table_1.DOCX]

| Antibody | Species | Company, Catalog # | Lot # | Concentration |
| --- | --- | --- | --- | --- |
| Acetylated tubulin  (6-11B-1) | mouse | Sigma-Aldrich, T6793 | 075M4860V | 1:500 (Whole mount)  1:4000 (Western Blot) |
| Adenylate cyclase 3 | rabbit | Invitrogen, PA5-35382 | TA2509452 | 1:500 (Whole mount)  1:3000 (FACS) |
| A cyclase III (C-20) | rabbit | Santa Cruz, sc-588, discontinued | C0615 | 1:100 (FACS) |
| Smoothened | rabbit | Novus Biologicals, NLS-2666 | 39584 | 1:100 |
| PDGFRa-*PE | rat | Invitrogen, 12-1401-81 | 4315814 | 1:100 |
| CXCR4-*PE | rat | Invitrogen, 12-9991-82 | 4344404 | 1:200 |
| Prominin-1-*APC | rat | Miltenyi Biotec, 130-102-197 | 5180605657 | 1:100 |
| Prominin-1 | rat | Gift from Denis Corbeil, TU Dresden | N/A | 1:70 |
| β-catenin (H-102) | rabbit | Santa Cruz, sc-7199 | ?? | 1:100 |
