# Supplementary material for: A Flow Cytometry-Based Approach for the Isolation and Characterization of Neural Stem Cell Primary Cilia
Source: Front Cell Neurosci. 2019 Jan 14;12:519. doi: 10.3389/fncel.2018.00519 (PMC6339872; doi:10.3389/fncel.2018.00519)
Supplement: FIGURE S2 — Sorting of particles according to size. Quantification of the relative abundance of sorted particles of different sizes in embryo (E18) 8 (8w) and 25 (25w) week-old mice from three independent experiments. Values represent the average percentage of particles in each population. [file Table_2.DOCX]

| Marker | Primary cilia | Ependymal motile cilia |
| --- | --- | --- |
| Prominin-1 | + | +++ |
| AC3 | +++ | - |
| Acetylated Tubulin | +++ | +++ |
| PDGFRα | +++ | - |
| CXCR4 | + | ? |
| Smoothened | +++ | - |
