# Supplementary figures and images for: A Flow Cytometry-Based Approach for the Isolation and Characterization of Neural Stem Cell Primary Cilia
Source: Front Cell Neurosci. 2019 Jan 14;12:519. doi: 10.3389/fncel.2018.00519 (PMC6339872; doi:10.3389/fncel.2018.00519)

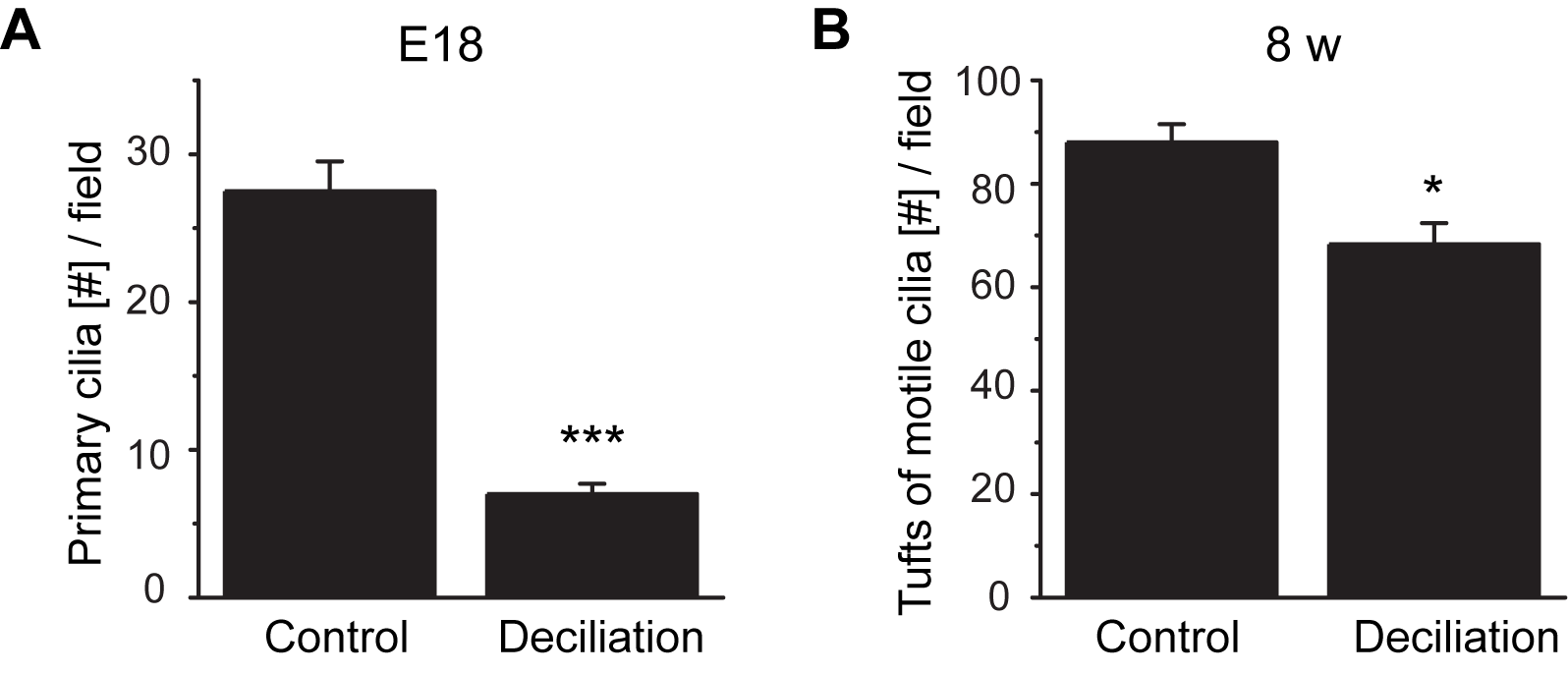

Supplement: TABLE S1 — List of antibodies used in this study. [file Image_1.TIF]

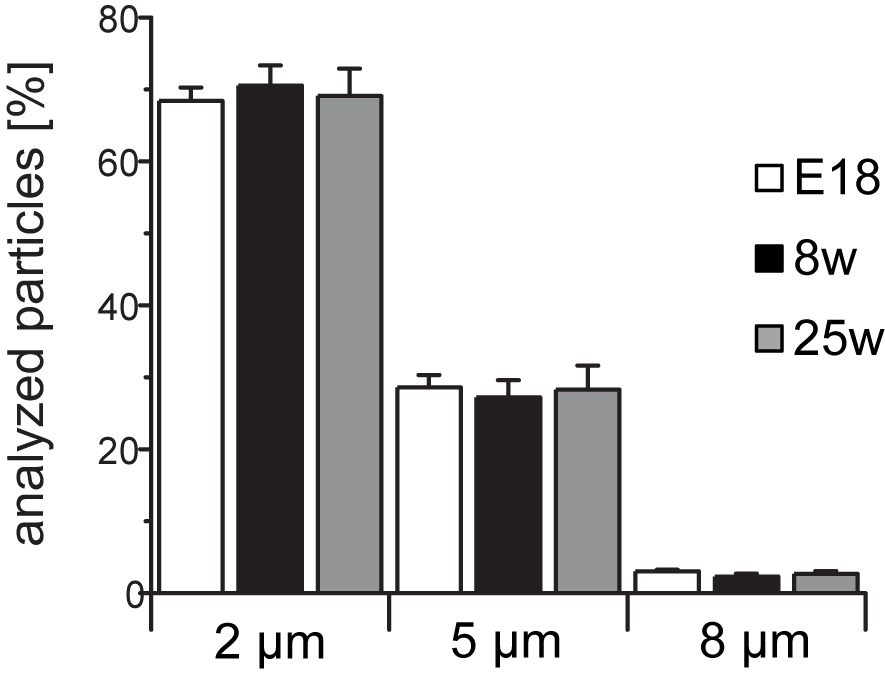

Supplement: TABLE S2 — List of markers used in this study to identify and characterize primary and motile cilia. [file Image_2.TIF]
